# Evaluating Patients’ Profiles and Procedural Outcomes of Robotically Assisted Percutaneous Coronary Interventions: A Single Centre Experience

**DOI:** 10.3390/jcm15155831

**Published:** 2026-07-25

**Authors:** Aleksander Zelias, Adriana Zlahoda-Huzior, Krzysztof Piotr Malinowski, Katarzyna Kolodziej-Zurawska, Magdalena Bogdan, Arif Khokhar, Michal Sobczyk, Ryszard Gajdosz, Jerzy Sadowski, Julia Zaber, Dariusz Dudek

**Affiliations:** 1 Faculty of Medicine and Health Science, University of Applied Science in Nowy Sacz, 33-300 Nowy Sacz, Poland; 2 2nd Clinical Department of Cardiology, Faculty of Medicine and Health Science, University of Applied Science in Nowy Sacz, 33-300 Nowy Sacz, Poland; 3 Simhub, Virmed, 31-514 Krakow, Polandjzaber@simhub.pl (J.Z.); 4 Department of Measurement and Electronics, AGH University of Science and Technology, 30-059 Krakow, Poland; 5 Centre for Digital Medicine and Robotics, Jagiellonian University Medical College, 31-034 Krakow, Poland; 6 The Heart Centre, Rigshospitalet, Copenhagen University Hospital, 2100 Copenhagen, Denmark

**Keywords:** R-PCI, robotically assisted percutaneous coronary intervention, CorPath GRX, patients’ selection, procedural outcomes

## Abstract

**Background/Objectives:** Robotic-assisted percutaneous coronary intervention (R-PCI) reduces occupational hazards, may enhance procedural precision and has demonstrated comparable efficacy to manually performed PCI (M-PCI). However, the switch from M-PCI to R-PCI represents a unique challenge and learning curve, which remains undefined. The purpose of this study was to evaluate patients’ profile and procedural outcomes over time after starting an R-PCI programme at a tertiary cardiac centre. **Methods:** All consecutive patients who underwent R-PCI with the second-generation CorPath GRX R-PCI system at our centre between March 2021 and January 2024 were included. The study cohort was divided into two equal groups representing the early and late experience. Clinical profile, lesion characteristics and procedural outcomes were compared between both groups. Angiographic success was defined as TIMI 3 flow and less than 30% stenosis, and R-PCI technical success as angiographic success without any unplanned manual input. **Results:** For 102 patients (mean age 68 years, 73% male) who underwent R-PCI, the angiographic success rate was 100%, and the R-PCI technical success rate was 83.33%, with 16.67% of patients requiring unplanned manual input. In the late (n = 51) versus early (n = 51) group, more bifurcations (52.94% vs. 33.33%, *p* = 0.046) and ACC/AHA (American College of Cardiology/American Heart Association) type C lesions (45.10% vs. 25.49%, *p* = 0.038) were treated with no significant differences in contrast volume, radiation dose, procedural time or peri-procedural complications between the two groups. **Conclusions**: R-PCI appears feasible with acceptable short-term results. In our experience, after ~50 cases, increasing complexity of lesions can be treated without significantly impacting procedural outcomes or safety.

## 1. Introduction

Coronary artery disease (CAD) represents one of the most significant contributors to global mortality and disability [1]. Percutaneous coronary intervention (PCI) has become the pillar of revascularization in patients with obstructive coronary disease [2]. Despite major advances in stent technology, imaging, and pharmacotherapy, PCI continues to expose operators to substantial occupational hazards, including cumulative radiation exposure and musculoskeletal injuries associated with prolonged use of heavy lead protection [3]. These challenges have stimulated interest in technological innovations that could improve both procedural precision and operator safety. Robotic-assisted percutaneous coronary intervention (R PCI), first demonstrated in 2004, was developed to address several limitations of manual PCI. Over the past two decades, R PCI has shown safety and efficacy in multiple large real-world registries [4,5,6,7], demonstrating its ability to enhance precision and reproducibility of intracoronary device manipulation [8,9]. By enabling operators to perform PCI from a radiation-free remote workstation, R-PCI substantially reduces radiation exposure and orthopedic strain—benefits that are increasingly relevant as interventional cardiologists face rising cumulative occupational risk [10]. However, the adoption of R-PCI has been limited due to compatibility constraints of early-generation robotic systems and the need for careful case selection during programme initiation, with procedural outcomes evolving over time as centres adapt to robotic workflows [11].

Switching from a bedside procedure that relies on direct manual manipulation of intracoronary equipment in a high-radiation environment to a system in which devices and catheters are controlled robotically from a radiation-free remote workstation fundamentally alters procedural workflow, case selection strategy, and the pattern of procedural outcomes observed during programme initiation.

Limited data exist describing how case selection evolves and how procedural outcomes change over time after the initiation of an R-PCI programme. Therefore, we conducted this study to evaluate patterns of case selection and procedural performance during the implementation of an R-PCI programme using the latest-generation CorPath GRX (Corindus, Siemens Healthineers, Erlangen and Munich, Germany) robotic platform.

## 2. Materials and Methods

This is an observational, retrospective study including consecutive patients who underwent R-PCI using the CorPath GRX 2nd-generation robotic platform (Corindus, Siemens Healthineers, Erlangen and Munich, Germany) in a single invasive cardiology centre (2nd Clinical Department of Cardiology Intercard, Nowy Sacz, Poland) between March 2021 and January 2024. The study was designed as a prospective registry with electronic medical data collection in the hospital database and planned 1-year follow-up using telephone contact. All patients signed informed consent for the intervention and data processing. We obtained formal approval of the local ethics committee (no. OIL/KBL/20/2025) for the publication of the study results.

### 2.1. Study Robot

**Figure 1:**
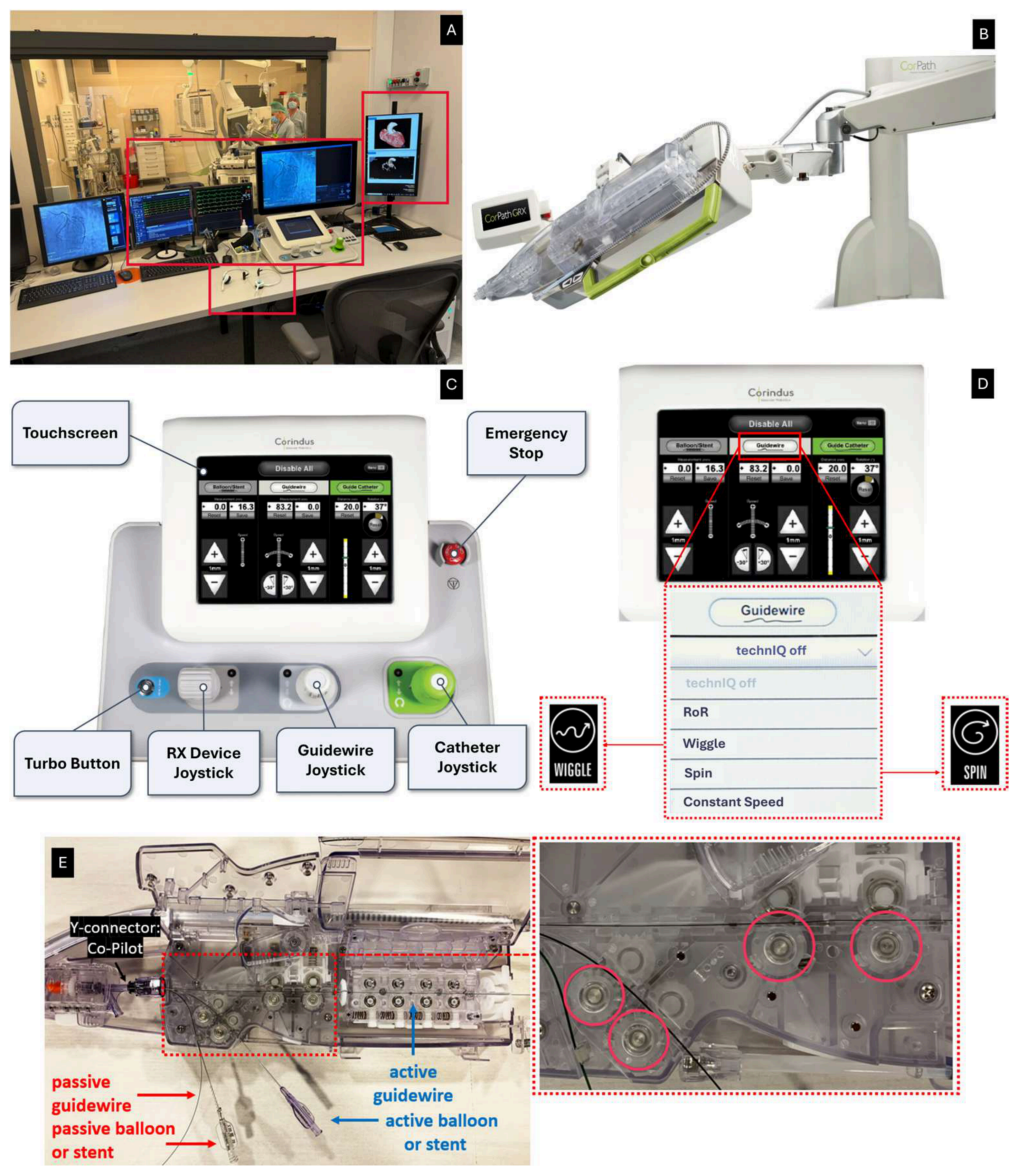
CorPath GRX system overview. (**A**) Overview of system installation; (**B**) Robotic arm with a cassette mounted on (**C**) Robotic console with joystick and touch screen controls; (**D**) Robotic automations (TechnIQ); (**E**) Close view of the cassette.

The CorPath GRX (Corindus, Siemens Healthineers, Erlangen and Munich, Germany) is a second-generation robotic system that consists of a robotic arm mounted at the patient’s bedside and a robotic cockpit, which can be positioned either inside or outside the cath lab. The robotic cockpit is equipped with three joysticks to separately control movements of the guide catheter (GC), coronary wire and coronary device (balloon or stent), each of which can also be controlled by a touchscreen interface. (Figure 1) Additional smart automation maneuvers, termed TechnIQ, allow common movements and manipulations of the coronary guidewire and devices to be replicated (i.e., wiggle, spin and rotate on retract for the wires and constant speed or dotter for balloons or stents).

### 2.2. Robotic Procedures

All robotic procedures were performed by two experienced operators together with a dedicated team of nurses and technicians trained and experienced with the robotic system. One operator was located inside the operating room and managed the access site, GC insertion and coronary equipment exchange inside the robotic cassette, with a second operator seated at the robotic console positioned outside the operating room remotely controlling the movement of the loaded devices. Bilateral audio-visual communication between the operators and auxiliary staff was maintained throughout using dedicated headsets and cameras.

### 2.3. Study Protocol

There were no prespecified inclusion criteria for R-PCI in our institution. Conversely, eligibility for R-PCI was determined case by case by two experts trained in R-PCI procedures following review of the diagnostic coronary angiogram. PCI was deemed suitable for robotic intervention if both operators agreed that there was a high chance of achieving an optimal angiographic result with a low probability of requiring unplanned manual conversion.

In addition, we performed R-PCI when all dedicated cath lab team and both expert operators were available at the same time, so the procedures were done only on selected, prespecified days, instead of as an everyday routine. We excluded all STEMI patients to expedite immediate artery re-opening and chronic total occlusions (CTO) due to the limitation of not being able to robotically control microcatheters and lack of tactile feedback.

Data on patients’ demographics, past medical history, lesion characteristics (location, bifurcation, ostial coronary involvement and lesion severity according to ACC/AHA classification [12], procedural characteristics and efficacy measures (contrast volume, radiation dose, procedural and fluoroscopy time), periprocedural complications (including flow-limiting dissections, acute vessel or side branch closure, periprocedural myocardial infarction or stroke, access side hematoma or major bleeding, defined as ≥3 according to Bleeding Academic Research Consortium—BARC criteria [13]), as well as one year follow-up major adverse cardiovascular events (MACE), including death, stroke, myocardial infarction (MI), and target and non-target vessel (TVR) or lesion (TLR) revascularization, were collected using existing hospital electronic health data records and/or telephone contact with patients or their relatives in follow-up. Both periprocedural and late MI were diagnosed according to the 4th universal definition [14]. All events were adjudicated by the study personnel with no external core lab for data verification and validation. Procedural time was defined as the time from insertion of GC to removal of arterial access sheath.

Angiographic success was defined as residual diameter stenosis of the treated lesion <30% with TIMI 3 flow post-intervention. Technical success was defined as completion of R-PCI without any unplanned manual assistance or permanent conversion to manual PCI. In selected cases where kissing balloon inflation was deemed necessary due to lesion anatomy (true bifurcations), the so-called hybrid approach was allowed, with limited manual input required for delivery and positioning of the second balloon; however, this was not regarded as manual conversion.

To evaluate how patients’ profile and procedural outcome changed over time, we divided all R-PCIs done in our institution into two equally sized groups: the first 50% of cases (early experience), and the remaining 50% of patients treated thereafter (late experience).

## 3. Statistical Analysis

Categorical variables were presented as numbers and percentages, and continuous variables as means with standard deviation or median with the first and the third quartile, for normally and not normally distributed variables, respectively. Normality of distribution was tested using the Shapiro–Wilk test. Normally distributed variables were compared between groups using Student’s *t*-test, and not normally distributed variables using the Mann–Whitney U test. Nominal variables were compared using the χ^2^ test and Fisher’s exact test. All statistical analyses were done using R version 4.4.1 (The R Foundation for Statistical Computing, Vienna, Austria, 2024) software.

## 4. Results

### 4.1. Baseline Characteristics

**Figure 2:**
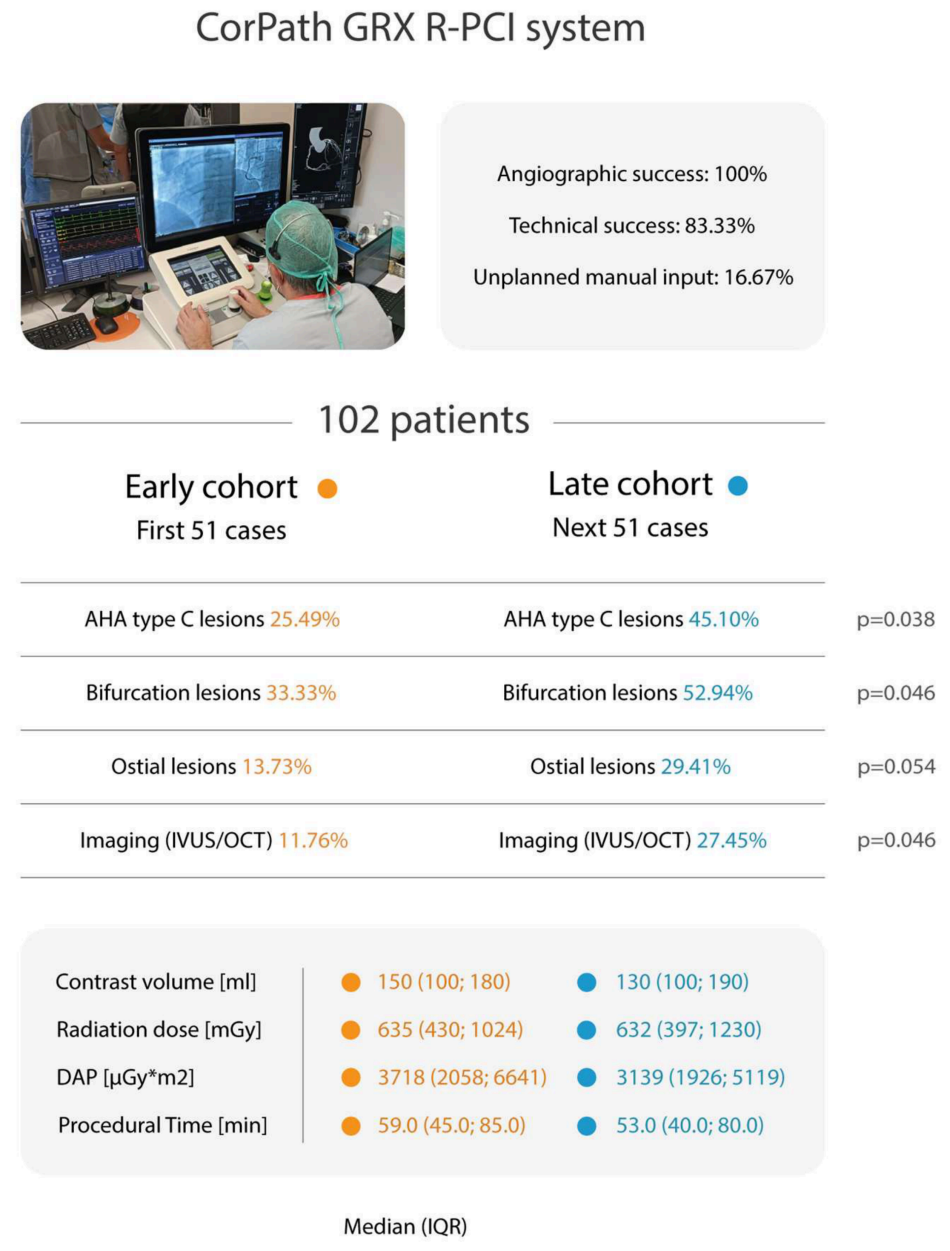
Central Illustration.

Between March 2021 and January 2024, 102 out of 1438 PCIs were performed using the robotic system (7.1%). However, in that time span, we performed R-PCI in only 63 separate operative days, and overall PCI volume on these days was 220, so the true proportion of cases selected for R-PCI versus total screened patients was 46% (102/220). For this study, 51 patients were included in the early phase and 51 patients in the late phase (Figure 2). Baseline characteristics are presented in Table 1. Mean age was 68.0 (±8.65) years; 73.5% were male, and the majority had a previous history of hypercholesterolemia (90.2%), hypertension (89.2%) and diabetes (52.9%). The majority of interventions were elective (91.2%) for chronic coronary syndromes, with a high percentage of patients (70.6%) having had previous PCI. Baseline characteristics were evenly distributed between the groups, except for the history of diabetes and previous PCI, which were more prevalent in the early phase and also had a lower median EF.

### 4.2. Lesion Characteristics

Across the entire cohort, left anterior descending, circumflex and right coronary lesions were equally treated. 68.6% of lesions were characterized as B2/C, with 43% bifurcation lesions and 21.6% ostial lesions. There was no difference in the treated vessel territories between the groups, but there were more type C lesions (45.1% vs. 25.5%; *p* = 0.038) and more bifurcations (52.9% vs. 33.3%; *p* = 0.046) in the late phase. Ostial locations were also numerically more frequent in the experienced phase but without statistical significance (29.4% vs. 13.7%; *p* = 0.054), as shown in Table 2.

### 4.3. Procedural Characteristics

Procedural characteristics are summarized in Table 3.

Radial access was widely utilized (94.1%) with 6Fr (86.3%) versus 7Fr (13.7%) GC preferred. The commonest catheter shape used was an Extra Back Up (EBU) for the left coronary artery (LCA) and Amplatz Right (AR) for the right coronary artery (RCA), with no significant differences between the groups. The need to exchange GC was also similar between the two groups. The average number and length of stents used was similar between the two groups, with numerically more bifurcation PCI performed during the late phase. Invasive functional assessment (FFR) interrogation was similar between the two groups, but there was increased use of intracoronary imaging (IVUS or OCT) and cardiac CT pre-planning in the late phase.

### 4.4. Procedural and Clinical Outcomes

Unplanned manual assistance occurred in 17 patients (16.7%), resulting in an overall R-PCI technical success rate of 83.3% without any difference between the two phases. Unplanned manual assistance was required for: lesion crossing (n = 7), treat coronary dissection with limited distal flow (n = 3), GC re-adjustment due to dampening of aortic pressures (n = 2) or unstable and non-coaxial alignment (n = 2), need for use of GC Extension due to inability to deliver a stent (n = 2) and robotic system failure (n = 1). Out of 17 cases of unplanned manual conversion, in only 5 cases (4.9%), 3 in the early and 2 in the late group, the conversion to manual PCI was permanent. In addition, in 10 cases (9.8%), 4 in the early and 6 in the late group, we used a hybrid approach, so the final number of true fully robotic procedures was 75 (73.53%), as shown in Table 4.

Despite more complex lesions being treated in the late phase, there was no significant difference in contrast volume (early phase: 150 mls vs. late phase: 130 mls, *p* = 0.68), radiation dose (early phase: 635 mGy vs. late phase: 632 mGy, *p* = 0.88), fluoroscopic time (early phase: 14.9 min vs. late phase: 15.8 min, *p* = 0.84) and procedural time (early phase: 59.0 min vs. late phase: 53.0 min, *p* = 0.26), as shown in Table 4.

There was a numerically higher rate of peri-procedural complications observed in the early (15.7%) compared to the late (7.8%) phase, although this did not reach statistical significance. These included 4 PCI-related (type 4a) MI, 3 flow-limiting dissections successfully stented without release of troponins, 3 access site hematomas, 1 periprocedural stroke and 1 iatrogenic Sinus of Valsalva dissection treated conservatively. We did not observe any major bleeding event according to BARC criteria. All complications were successfully managed with a final angiographic success rate of 100%. We obtained 1-year follow-up data for all studied patients. At 1-year MACE occurred in 12 patients (7 in the early and 5 in the late group) and consisted of: 4 deaths (3.92%)—2 due to heart failure, one related to stoke, and 1 non-cardiac, 7 unplanned PCI (6.86%) without MI (5 TVR/TLR, 2 non-TVR/TLR), and 1 MI due to target lesion failure but witout PCI (0.98%), as shown in Table 4.

## 5. Discussion

In this single-centre retrospective analysis of all consecutive patients who underwent R-PCI using a second-generation robotic platform we were able to demonstrate that: (1) after treating 100 patients with R-PCI, we were able to achieve high levels of procedural success (100%) and technical success (83.3%), (2) after ~50 cases, increasingly more complex B2/C and bifurcation lesions were treated with R-PCI, without a significant increase in the procedural time, contrast dose or radiation dose, and (3) a low rate of peri-procedural complications was observed.

This highlights that starting an R-PCI programme from scratch is feasible and can be successfully achieved within 50 cases for simple PCI, and within 100 cases for treating more complex lesions. We previously demonstrated that R-PCI can be done successfully in very complex cases, even for left main stenosis and for complex bifurcations with an upfront two-stent strategy [15,16]. But most of the time, especially in the early phase, we chose rather simpler and less demanding anatomies. With gained experience, we broadened patients’ categories, but always paid attention to safety, being prepared for immediate conversion to manual control in case of complications [17].

Patient selection is a key consideration when starting an R-PCI programme. In our early phase, there were more patients who had undergone previous PCI. This likely reflects the preference for elective second-stage procedures where the coronary anatomy was already known. Later, we more often decided ad hoc to proceed with R-PCI immediately after completion of diagnostic coronary angiography. Although no major haemorrhagic complications were observed in our study, careful assessment of a patient’s potential high bleeding risk (HBR) profile remains essential when planning any PCI, with procedural strategies adapted accordingly [18].

There are only a few reports describing how procedural outcomes change with time after the implementation of an R-PCI programme. Single-centre experience on 546 robotic cases (study period 2017–2020) [19] showed that crossovers to traditional PCI occurred only in the first half of procedures. Procedure time decreased until the first 50 cases and flattened thereafter. Similarly, contrast volume decreased with experience, with the slope flattening at procedure number 30 and fluoroscopy time demonstrated even faster shortening and levelled after the first 15 cases. No reduction in radiation dose was reported. Importantly, Syntax Score in these patients was very low (median 8 points), with no indication of increasing with gained experience. Accordingly, median fluoroscopy and procedural time in the R-PCI cohort was only 4.5 and 27 min and was much lower than in our study (15.0 and 55.0 min, respectively). They used less contrast volume (110 mL vs. 150 mL), but a similar radiation dose (698 mGy vs. 633 mGy). Moreover, the authors matched the results of R-PCI with M-PCI done in their institution and showed that the latter group had more complex anatomy (higher Syntax Score), was older and had lower ejection fraction. Therefore, the study results are probably applicable only to low-risk R-PCI and hence are contradictory to our observation that contrast volume and radiation dose, as well as fluoroscopy and procedural time, do not change with experience, presumably due to higher complexity of the later cases.

Another report from a large tertiary centre on establishing a successful robotic programme using the CorPath GRX system (study period 2021, 71 patients included, 88.4% with type B2/C lesions) indicates the overall clinical (angiographic) success rate was 100% and partial manual assistance was needed in 25.6%, while permanent conversion to manual occurred in only 5.8%. There were 7% periprocedural complications (4 MI’s and 1 stroke). Mean fluoro time was 20.4 min, procedural time 51.5 min, total contrast volume 145 mL and DAP 2298 μGy × m^2^—results similar to ours, except for seemingly less radiation dose in the cited study [20].

Other prospective single-centre experience on establishing an R-PCI programme using the CorPath GRX system recruited 83 patients between 2019 and 2020 with relatively complex anatomy (77.7% type B2/C lesion, 19.6% true bifurcations, 18.8% moderate to severe calcifications) and reported angiographic success in 99.1% and 86.6% technical success of robotic procedure (defined as absence of any unplanned manual conversion). The reason for manual assistance was mainly due to inability to advance wire, balloon or stent until or across the lesion or clinical condition of the patient, which required rapid medical intervention. These results are similar to our findings. Moreover, the authors compared their first 42 and last 41 patients and could not detect any differences in the profile of the patients and results of the procedures between the groups and, surprisingly, the procedural time was even longer in the late group (97.4 ± 57.6 vs. 75.9 ± 37.0, *p* = 0.046). The possible explanation of this finding, according to the authors, was their highly experienced team of interventionalists, nurses and technicians who rapidly embraced R-PCI, which damped the differences in procedural outcomes over time [21].

Another study focusing on R-PCI for complex coronary lesions (CORA-PCI) demonstrated a 99.1% angiographic and 91% technical success rate on 157 coronary lesions, 78.3% of type B2/C [22].

Indeed, the overall success rate of R-PCI procedures in the current literature is high and close to 100%, if partial manual assistance is not interpreted as robotic intervention failure. In a recent (2024) registry of 70 prospective R-PCIs performed with the CorPath GRX platform in a single university centre, matched with 70 manual PCI (M-PCI) and with comparable clinical and angiographic characteristics to our cohort, the success rate of R-PCI was 100% with the need for manual assistance in 19%, which is very similar to our results. Moreover, the one-year follow-up MACE rate in the R-PCI group (death—2.9%, unplanned PCI—5.7%, target vessel failure—2.9%) was again similar to our study. In addition, there were no difference regarding success and complication rates between R-PCI and M-PCI, although R-PCI were characterized with increased procedural (median 103 min vs. 67 min, *p* < 0.001) and fluoroscopy time (18 min vs. 15 min, *p* = 0.002) and more contrast volume used (180 mL vs. 160 mL, *p* = 0.041), without difference in radiation dose (dose-area product 4062 vs. 3242 cGycm2, *p* = 0.361) [23].

Finally, we would like to stress the importance of training nursing and overall staff for the successful implementation of robotic procedures. Specific positioning of the patient on the operating table equipped with a robotic arm, loading and exchanging interventional equipment to the robotic cassette requires adoption of specific routines and skills and would take some time to achieve smooth and swift performance, which may considerably influence procedural time. However, as was already mentioned, the procedural time in our study did not change in late vs. early procedures probably because the complexity of PCI performed increased. Another important aspect to be mentioned is the ability of audio-visual contact, esp. after moving an interventional cockpit outside the operating room. In our cath-lab, all interventional staff used headphones to allow undisturbed bilateral communication, and in addition there were cameras that transmitted video from the operating room to the screen positioned beside the robotic console for the main operator to maintain constant visual control on the patient status.

## 6. Limitations

Our study is limited by its single-centre, observational nature and the lack of external adjudication of the events. There were no prespecified inclusion criteria for R-PCI case selection, and thus they were subjective and changed over time; however, one of the aims of our study was to describe how patients’ profile differed between early and late cases. CorPath GRX platform used in the study does not allow simultaneous active control of two wires or two balloons and does not officially support mechanical (rotational) IVUS/OCT probes, as well as GC extension or microcatheter, which may have influenced patient selection. The recruitment was also restricted by lack of reimbursement for R-PCI procedures in Poland and unexpected withdrawal of Siemens from supporting the CorPath GRX platform. In addition, due to overall low case volume, there were only two expert operators trained and performing R-PCI, which limits generalization of the results. Likewise, due to the limited number of patients, the procedural complications and manual conversion rates were too few to build a reliable multivariate prediction model. Increased use of intracoronary imaging and CT-pre-procedural planning over time may have influenced observed procedural outcomes, but the difference was not statistically significant. Moreover, we did not perform formal learning curve analysis using dedicated statistical methods. In addition, we did not compare the results of R-PCI with M-PCI done in our institution, calculate Syntax Score or assess calcium severity and lesion tortuosity.

## 7. Conclusions

The results of our study indicate that the adoption of R-PCI procedures for already skilled operators appears feasible in a short time with acceptable short-term results and with similar safety and efficacy of the early and late interventions. We could not detect any difference in procedural and fluoroscopy time, contrast volume or radiation dose. However, the later cases were more complex with more bifurcations and type C lesions and more often intracoronary imaging, but, on the other hand, with a tendency for more frequent manual assistance.

In our opinion, before R-PCI become more widely used, it needs to offer simultaneous control of two wires and balloons, better control of the position of the guide catheter and the possibility to manipulate micro- or guide extension catheters. Current technology, although proven to be safe in experienced hands, still requires frequent manual aid by an assistant operator on the patient side.

## Figures and Tables

**Table 1:** Baseline patient characteristics.

Variable	First Half	Second Half	Total	*p*
N	51	51	102	
Male	36 (70.59)	39 (76.47)	75 (73.53)	0.501
Age [years]	68.98 (±8.39)	67.08 (±8.89)	68.03 (±8.65)	0.269
BMI [kg/m^2^]	28.22 (24.89; 29.82)	28.23 (26.17; 31.05)	28.23 (25.87; 30.51)	0.463
Hypertension	46 (90.20)	45 (88.24)	91 (89.22)	0.759
Dyslipidemia	46 (90.20)	46 (90.20)	92 (90.20)	>0.99
Current smoking	17 (33.34)	23 (45.10)	40 (39.22)	0.427
GFR [mL/min/1.73 m^2^]	66.12 (±17.55)	74.48 (±24.70)	70.30 (±21.73)	0.051
Diabetes	32 (62.75)	22 (43.14)	54 (52.94)	0.047
Previous MI	17 (33.33)	13 (25.49)	30 (29.41)	0.385
Previous PCI	42 (82.35)	30 (58.82)	72 (70.59)	0.009
2nd stage PCI	33 (64.71)	18 (35.29)	51 (50.00)	0.029
Previous CABG	1 (1.96)	2 (3.92)	3 (2.94)	>0.99
PAD	9 (17.65)	15 (29.41)	24 (23.53)	0.161
Stroke	2 (3.92%)	3 (5.88%)	5 (4.90%)	>0.99
EF [%]	0.55 (0.45; 0.60)	0.58 (0.50; 0.65)	0.55 (0.47; 0.63)	0.035
Acute presentation	4 (7.84)	5 (9.80)	9 (8.82)	>0.99

Results are presented as numbers and percentage for categorical and as mean (+/−SD) or median and interquartile range (Q1; Q3) for continuous variables depending on normality; BMI—body mass index; GFR—glomerular filtration rate; MI—myocardial infraction; PCI—percutaneous coronary intervention; CABG—coronary artery bypass grafting; EF—ejection fraction; acute presentation—unstable angina or non ST elevation myocardial infarction.

**Table 2:** Lesions characteristics.

Variable	First Half	Second Half	Total	*p*
N	51	51	102	
R-PCI treated vessel				0.325
LM	3 (5.88)	4 (7.84)	7 (6.86)	
LAD/DG	15 (29.41)	22 (43.14)	37 (36.27)	
CX/MG	20 (39.22)	12 (23.53)	32 (31.37)	
RCA/RPD	13 (25.49)	13 (25.49)	26 (25.49)	
Lesion severity [ACC/AHA]				0.021
A	1 (1.96)	5 (9.80)	6 (5.88)	
B1	18 (35.29)	8 (15.69)	26 (25.49)	
B2	19 (37.25)	15 (29.41)	34 (33.33)	
C	13 (25.49)	23 (45.10)	36 (35.29)	
Type C lesion [ACC/AHA]	13 (25.49)	23 (45.10)	36 (35.29)	0.038
Ostial location	7 (13.73)	15 (29.41)	22 (21.57)	0.054
Bifurcation lesion	17 (33.33%)	27 (52.94%)	44 (43.14%)	0.046

Results are presented as numbers and percentage for categorical and as mean (+/−SD) or median and interquartile range (Q1; Q3) for continuous variables depending on normality; LM—left main; LAD—left anterior descending; DG—diagonal; CX—circumflex; MG—marginal; RCA—right coronary artery; RPD—right posterior descending artery; ACC/AHA—American College of Cardiology/American Heart Association.

**Table 3:** Procedural characteristics.

Variable	First Half	Second Half	Total	*p*
N	51	51	102	
Access				>0.99
Femoral	3 (5.88)	2 (3.92)	5 (4.90)	
Radial	48 (94.12)	49 (96.08)	97 (95.10)	
GC French size				>0.99
6	44 (86.27)	44 (86.27)	88 (86.27)	
7	7 (13.73)	7 (13.73)	14 (13.73)	
Non-Judkins GC	42 (82.35)	43 (84.31)	85 (83.33)	0.79
Need for exchange of GC	6 (11.76)	5 (9.80)	11 (10.78)	0.74
KBI and/or SBI	6 (11.76)	9 (17.65)	15 (14.71)	0.40
No of stents per case	1.0 (1.0; 2.0)	1.0 (1.0; 2.0)	1.0 (1.0; 2.0)	0.18
Total stent length [mm]	33 (23.0; 41.0)	28 (16.0; 44.0)	28 (23.0; 43.3)	0.17
2-stent technique	1 (1.96)	4 (7.84)	5 (4.90)	0.36
FFR	6 (11.76)	11 (21.57)	17 (16.67)	0.18
Imaging (IVUS/OCT)	6 (11.76)	14 (27.45)	20 (19.61)	0.046
CCTA pre-procedural planning	0 (0.00)	15 (29.41)	15 (14.71%)	<0.01

Results are presented as numbers and percentages for categorical and as mean (+/−SD) or median and interquartile range (Q1; Q3) for continuous variables depending on normality; GC—Guide Catheter; Non-Judkins GC = Extra Back Up/EBU/or Amplatz type catheter; KBI/SBI—kissing balloons/side branch inflation; IVUS—intravascular ultrasound; OCT—optical coherent tomography; FFR—fractional flow reserve; CCTA—cardiac computed tomography angiography.

**Table 4:** Procedural and clinical outcomes.

Variable	First Half	Second Half	Total	*p*
N	51	51	102	
Contrast volume [mL]	150 (100; 180)	130 (100; 190)	150 (100; 180)	0.68
Radiation dose [mGy]	635 (430; 1024)	632 (397; 1230)	633 (419; 1167)	0.88
DAP [μGy × m^2^]	3718 (2058; 6641)	3139 (1926; 5119)	3387 (1990; 5676)	0.15
Fluoroscopy time [min]	14.9 (10.3; 23.5)	15.8 (9.9; 24.3)	15.0 (10.3; 24.0)	0.84
Procedural time [min]	59.0 (45.0; 85.0)	53.0 (40.0; 80.0)	55.0 (42.5; 80.0)	0.26
Periprocedural complications	8 (15.69)	4 (7.84)	12 (11.76)	0.22
Unplanned manual conversion	7 (13.73)	10 (19.61)	17 (16.67)	0.43
Permanent manual conversion	3 (5.88)	2 (3.92)	5 (4.90)	0.65
Hybrid approach (for KBI)	4 (7.84)	6 (11.76)	10 (9.80)	0.51
True fully robotic procedure	40 (78.43)	35 (68.63)	75 (73.53)	0.26
Technical success of R-PCI	44 (86.27)	41(80.39)	85 (83.33)	0.43
Angiographic success	51 (100)	51 (100)	102 (100)	>0.9
1-year FU MACE	7 (13.7)	5 (9.8)	12 (11.76)	0.54

Results are presented as numbers and percentage for categorical and as mean (+/−SD) or median and interquartile range (Q1; Q3) for continuous variables depending on normality; DAP—Dose aera product; R-PCI-robotic percutaneous coronary intervention; FU—follow-up; Angiographic success—TIMI 3 flow and residual stenosis <30%; Technical success of R-PCI—angiographic success without unplanned manual assistance or need of permanent discontinuation of robotic procedure; MACE—Major Adverse Cardiovascular Events; KBI—Kissing balloon inflation.

## Data Availability

The manuscript data are in possession of the authors and could be presented to the readers on request.
